# Carbohydrate-to-Fiber Ratio, a Marker of Dietary Intake, as an Indicator of Depressive Symptoms

**DOI:** 10.7759/cureus.17996

**Published:** 2021-09-15

**Authors:** Sarah S Makhani, Camron Davies, Kevin A George, Grettel Castro, Pura Rodriguez de la Vega, Noel C Barengo

**Affiliations:** 1 Department of Medical and Population Health Sciences Research, Herbert Wertheim College of Medicine, Miami, USA; 2 Department of Public Health, University of Helsinki, Helsinki, FIN

**Keywords:** depression, carbohydrates, fiber, dietary fiber, nhanes

## Abstract

Objective: The aim of this study is to evaluate the association between a marker of dietary intake, the carbohydrate-to-fiber (CF) ratio, and moderate-to-severe depressive symptoms.

Design: Cross-sectional study.

Setting: National Health and Nutrition Examination Survey (NHANES) database from 2013-2016.

Participants: Individuals 18 years and older were included. Participants with total energy intake outside of three standard deviations of the mean, pregnant or breastfeeding women, and those with missing data were excluded.

Measurements: The main independent variable, CF ratio, was generated using corresponding variables in NHANES and divided into quartiles. The main outcome was depressive symptoms using the Patient Health Questionnaire-9. Unadjusted and adjusted logistic regression analyses were used to calculate odds ratios and their corresponding 95% confidence interval (CI).

Results: Among all participants (n=9,728), 8.3% reported to have moderate-to-severe depressive symptoms (n=833). The highest proportion of depressive symptoms was reported in respondents in quartile 4 (Q4), with the highest CF ratio (13.0%; p<0.001). After adjustment, the odds of depressive symptoms significantly increased in Q4 of the CF ratio compared with Q1 (adjusted odds ratio 1.4, 95% CI 1.0-1.9). The prevalence of depressive symptoms significantly increased in females, lower federal poverty levels, non-married individuals, smokers, and hypertension patients.

Conclusion: This nationally representative sample suggests that a higher CF dietary intake ratio increases the risk of moderate-to-severe depressive symptoms. These results suggest that the CF ratio may help clinicians and patients evaluate their dietary risk for depressive symptoms. Further prospective studies are needed to validate this ratio as a dietary measurement.

## Introduction

From 2013-2016, an estimated 8.1% of adults in the United States had Major Depressive Disorder (MDD), making it one of the most common psychiatric disorders in the United States (US) [1]. A majority of those with MDD report significant impairments at work, home, or with social activities [2]. Moreover, MDD increases the risk of chronic diseases, productivity loss, and suicide [2]. Given these widespread and diverse effects, it is necessary to identify modifiable risk factors for depressive symptoms that could lead to MDD and potential preventative measures.

In addition to the current standards of care, one of the most promising avenues for intervention is via the gut microbiome and gut-brain axis (GBA). Intestinal microflorae have the ability, through digestion and fermentation of the foods, to produce a vast range of neuroactive and cell signaling molecules such as short-chain fatty acids (SFCA), acetylcholine (ACh), catecholamines, gamma-aminobutyric acid (GABA), histamine, melatonin, and serotonin. The gut produces almost 90% of the body’s serotonin [3]. Moreover, SFCAs like acetate and butyrate play vital roles in regulating the immune system. For example, butyrate is highly involved in maintaining the integrity of the intestinal epithelium and inducing regulatory T-cells. Decrease in butyrate can lead to generalized inflammation through a variety of mechanisms, including increased nuclear factor- κB (NF-κB) synthesis and lipopolysaccharide (LPS) absorption, which in turn can increase the permeability of the blood-brain barrier [4]. This can lead to neuroinflammation, generalized sickness behavior, and even MDD [5]. Notably, a low intake of dietary fiber was found to be a major limiting factor for maintaining a viable and diverse microbiota and production of SCFAs in the gut [4]. It has been demonstrated that dietary fibers with prebiotic properties can modulate host gene expression and metabolism [6]. Moreover, it has also been shown that acellular flours, sugars, and processed foods, in other words, high-glycemic and high-carbohydrate foods, produce an inflammatory microbiota [7]. 

Given this putative pathology, it is plausible that the gut-brain axis (GBA), as mediated by nutrition, may have an impact on mental health. Indeed, preliminary evidence suggests that nutrient intake correlates with mental health outcomes [8,9]. Specifically, total fiber has been found to be inversely associated with the prevalence of depressive symptoms and carbohydrate intake has been shown to be associated with depression [10,11]. Accordingly, the pro-inflammatory and anti-inflammatory balance between carbohydrates and fiber may be more important than the individual nutrients alone. Thus so far, despite evidence implicating carbohydrate and fiber intake in the shared pathogenesis of MDD, no studies have combined the two measurements into one metric. Compared to individual measures of carbohydrates or fiber, the carbohydrate-to-fiber (CF) ratio paints a more holistic picture of one’s diet; a high ratio may indicate an increased intake of processed or refined foods, while a lower ratio may indicate a diet of more whole or unprocessed foods. In turn, given the diet’s links with mood, the CF ratio may correlate with varying degrees of depressive symptoms.

The objective of this study was to investigate whether depressive symptoms may be positively associated with one’s dietary CF ratio. The hypothesis is that as the CF ratio increases, there will be an increase in the prevalence of depressive symptoms related to the pro-inflammatory effects of refined carbohydrates and the protective effects of fiber.

## Materials and methods

Study design and data source

This is a cross-sectional study using secondary data from the National Health and Nutrition Examination Survey (NHANES) database from years 2013-2016. The NHANES database aims to determine the health and nutritional status of both children and adults in the United States. The nationally representative data is collected by conducting household visits with a team of physicians, dietary interviewers, and health technicians. NHANES includes a combination of self-reported lifestyle choices and objective medical data collected by the professionals during the visit. This well-recognized and nationally validated database is used to determine lifestyle implications and risk factors on one’s health outcomes [12]. This survey examines a sample of approximately 5,000 persons per year, who are widely distributed across the country. The interview involves questions regarding demographic, dietary, and socioeconomic factors. Many of the staff members are bilingual, reducing possible translation barriers. The examination includes medical and physiological measurements and laboratory tests. The dietary interview component of NHANES called ‘What We Eat in America’, is conducted as a partnership between the U.S. Department of Agriculture (USDA) and the U.S. Department of Health and Human Services (DHHS) [13]. DHHS is responsible for the survey design and data collection, and the USDA is responsible for the data collection methodology, maintenance of the databases used to code and process the data, and data review and processing. All NHANES participants were eligible for two 24-hour dietary recall interviews. The first dietary recall interview, which was collected in-person, was used for the data on carbohydrate and dietary fiber intake for this study. Due to the study design, this study was exempted by the governing Institutional Review Board.

Study population

**Figure 1:**
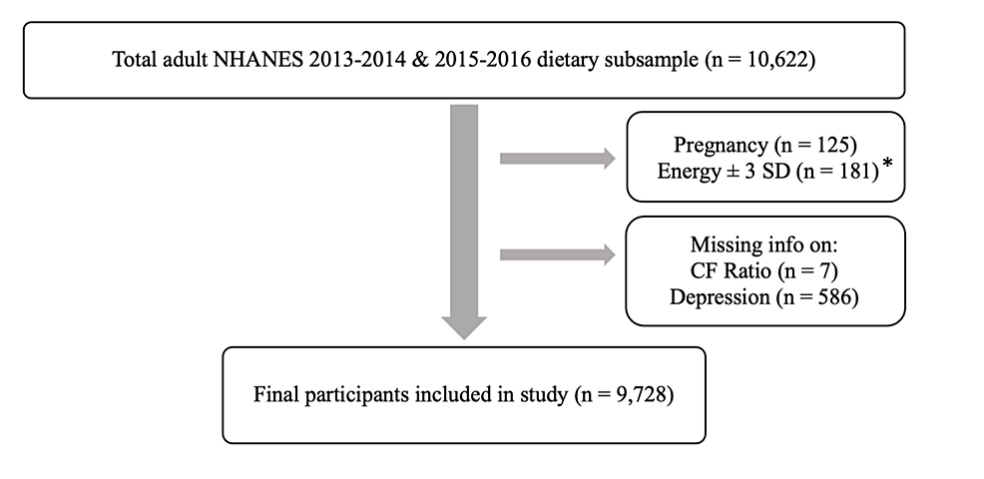
Flow chart showing exclusion criteria. Energy +/- SD: Total caloric intake above or below 3 standard deviations; CF Ratio: Carbohydrate-to-fiber Ratio

This subsample included adults 18 years of age and older who participated in the NHANES survey between the years 2013-2016 (Figure 1). This age range was selected as NHANES only reports Patient Health Questionnaire-9 (PHQ-9) data for individuals 18 years old and older. The exclusion criteria for this study included the following: pregnant and/or breastfeeding women and individuals with missing information on any of the key variables. Pregnant and/or breastfeeding women were excluded as the dietary intake may have been altered and inconsistent with the individual's baseline intake. 

Carbohydrate-to-fiber ratio

The ratio of carbohydrate-to-fiber (CF) dietary intake was the independent variable, as measured by a validated questionnaire. The CF ratio was generated using the daily carbohydrate intake (in grams) divided by the daily dietary fiber intake (in grams). This measure was further categorized into quartiles (2.0-12.0, 12.1-16.5, 16.6-23.0, 23.1-724.4). The first quartile was then chosen as the reference category given that the USDA recommendation for daily intake is 30g of fiber and 225-325g of carbohydrate, based on a 2000 calorie diet, which fell into the first quartile of this variable [14].

Depressive symptoms

The dependent variable of this study was depressive symptoms and the PHQ-9 questionnaire was used to assess the severity of the depressive symptoms and the likelihood that the patients would meet the criteria for MDD (i.e. clinical depression). The PHQ-9 questionnaire is a validated screening tool that includes questions about different areas of mental health. The 9 items on the PHQ‐9 consist of criteria used by the Diagnostic and Statistical Manual of Mental Disorders, Fifth Edition (DSM‐V) to diagnose a major depressive disorder. Each item is ranked from 0 (“not at all”) to 3 (“nearly every day”), with the total score ranging from 0 to 27. PHQ-9 scores greater than 5, 10, 15, and 20 represent mild, moderate, moderately severe, and severe depression, respectively [15]. Given that a PHQ-9 score greater than or equal to 10 has a sensitivity of 88% and a specificity of 88% for major depressive disorder [15], a PHQ-9 score of 10 was chosen as the cutoff value for determining moderate-to-severe depressive symptoms. Accordingly, this study used a dichotomous variable for baseline and statistical analysis: PHQ-9 scores equal to or greater than 10 represented moderate-to-severe depressive symptoms, while PHQ-9 scores less than 10 represented the absence of symptomatic depression.

Covariates

Based on previous literature, the following variables were included to control for the potential effects of confounding [16]. Demographic information included: sex; age divided into 18-39 years, 40-59 years, and ≥ 60 years; and race categorized as Mexican American, other Hispanic, non-Hispanic White, non-Hispanic Black, non-Hispanic Asian, and other races. Social factors that were accounted for included: marital status categorized as married, living with a partner, or not married; educational level categorized as below high school, high school, and above high school; smoking status defined as smoking at least 100 cigarettes in life or not; and alcohol consumption status defined as non-drinkers, moderate drinkers, and heavy drinkers. Non-drinkers were determined as participants who reported drinking less than 12 drinks per year; moderate drinkers as participants who reported consuming one drink per day for women and two drinks per day for men; and heavy drinkers as participants who reported drinking more than one drink per day for women and more than two drinks per day for men. Socioeconomic status was assessed using the poverty income ratio with groups divided into less than 130%, 131-250%, and 251-500% of the federal poverty line; 130% was chosen as the main cutoff as this is the inferior limit to receive Supplemental Nutrition Assistance Program (SNAP) [17]. Physical factors included body mass index (BMI) measured as kg/m^2^ and categorized according to the Center for Disease Control and Prevention (CDC) criteria into normal weight (<25), overweight (25 to < 30), and obese (≥ 30.0); physical activity as measured by the hours per day of moderate and vigorous work activity and recreational activity; and total energy intake measured by kilocalories consumed [18]. Physical activity was standardized across different intensity levels by calculating the metabolic equivalent (MET) hours per day, incorporating both moderate and vigorous activity. This measurement was calculated by multiplying the duration of vigorous and moderate physical activity per day by its corresponding MET score as described in NHANES [13]. Chronic medical conditions accounted for include hypertension and type II diabetes [19]. Patients that reported pre-diabetes were not included in the diabetes category.

Statistical analysis

Data analysis was performed using Stata 15.0 (StataCorp. L.L.C. College Station, TX, USA). Sampling weights were applied to generate nationally representative estimates. Baseline characteristics were presented as count and percentage (%) for categorical variables and by means and standard deviation (SD) for variables that were normally distributed; otherwise, median and interquartile range were calculated. Bivariate analysis was performed to identify the association of potential confounders with exposure and outcome variables. Percentages comparisons across categorical variables were performed using *X*^2^ tests. Linear univariate regression was performed to compare the mean levels of total energy intake across quartiles and between participants with and without moderate-to-severe depressive symptoms. Unadjusted and adjusted binary logistic regression analyses were conducted to evaluate the association between CF ratios and the presence of moderate-to-severe depressive symptoms, using the first quartile of the CF ratio as the reference. The multivariable regression model was adjusted for age, gender, race, education, BMI, alcohol consumption, poverty ratio, diabetes, hypertension, marital status, work and recreational physical activity, smoking, and total calorie intake. Odds ratios (OR) and their corresponding 95% confidence intervals were presented and a two-sided p-value of less than 0.05 was considered statistically significant. 

## Results

The baseline characteristics of the study participants according to the carbohydrate-to-fiber (CF) ratio (divided into quartiles) are presented in Table 1. Quartile 1 (Q1) included ranges 2.3-12.1, quartile 2 (Q2) included ranges 12.2-16.5, quartile 3 (Q3) included 16.6-22.9, and quartile 4 (Q4) included 23.0-724.4. Among participants from all quartiles (n=9,728), there was a higher proportion of respondents who identified as non-Hispanic white (p<0.001). Further, a higher frequency of all participants was married (p<0.001) and reported an education status greater than high school (p<0.001). Among racial minority groups, there was a higher proportion of African Americans in Q3 and Q4, while Mexicans and non-Hispanic Asians were more prevalent to be in Q1 (p<0.001). Quartile 1, deemed to be within the recommended range of CF ratio, contained more females (55.2%; p<0.001) and adults ranging from ages forty to fifty-nine (38.3%, p<0.001). Among people in this category, non-heavy drinkers (37.8%, p<0.001) and non-smokers (61.5%, p<0.001) had a higher frequency compared with their counterparts. Respondents in this category were also less likely to have diabetes (87.3%; p<0.001) and represented a socioeconomic status of over 250% of the federal poverty level (65.5%; p<0.001). Overall, the population in quartile one showed metrics and behaviors that are widely regarded to have healthier outcomes. In contrast, the higher quartiles showed a higher frequency of males (54.5%, p<0.001) and participants younger in age (46.2%; p<0.001). A majority of both Q3 and Q4 showed zero hours of recreational physical activity per day (p<0.001) and practices of heavy alcohol consumption (p<0.001). Additionally, those with the highest caloric intake were found in the highest carbohydrate to fiber quartile.

**Table 1:** Characteristics of study participants according to the carbohydrate-to-fiber ratio in the U.S. between 2013-2016. ^a^Metabolic equivalent

	Carbohydrate-to-Fiber Ratio Quartiles					
	Q1 2.3-12.1	Q2 12.2-16.5	Q3 16.6-22.9	Q4 23.0-724.4		
	(n=3047)	(n=2393)	(n=2114)	(n=2174)	p-value	
	%	%	%	%		
Age (years)					< 0.001	
18-39	29.2	37.3	40.3	46.2		
40-59	38.3	32.5	34.7	35.7		
60+	32.6	30.1	25.0	18.1		
Sex					0.002	
Male	44.8	48.9	49.4	54.4		
Female	55.2	51.1	50.5	45.6		
Race					<0.001	
Non-Hispanic Whites	67.2	67.2	63.1	63.2		
Mexican	11.2	9.3	8.5	7.0		
Other Hispanics	5.1	5.8	6.3	5.7		
Non-Hispanic African Am.	6.7	9.3	13.8	16.9		
Non-Hispanic Asians	6.8	5.6	4.6	3.3		
Other	3.1	2.8	2.8	4.0		
Education Level					<0.001	
Less than high school	12.1	13.1	14.7	18.4		
High school	15.6	21.5	26.0	30.5		
More than high school	72.3	65.3	59.3	51.1		
Poverty Ratio					<0.001	
1.30 and below	17.1	20.8	24.6	32.8		
1.3-2.5	17.4	21.7	23.8	24.4		
2.5-5.0	65.5	57.5	51.6	42.7		
Marital Status					<0.001	
Married	68.6	61.6	56.8	50.8		
Non-married	30.0	35.1	39.1	43.6		
N/A	1.4	3.32	4.03	5.59		
BMI (kg/m^2^)					0.078	
Under 25	30.8	27.9	27.8	29.1		
25 to <30	34.2	33.1	31.2	30.5		
30+	35.0	39.0	41.0	40.4		
Alcohol Intake					<0.001	
Less than 12 drinks/year	27.3	27.1	27.1	24.5		
Non-heavy drinkers	37.8	37.9	31.8	26.0		
Heavy drinkers	35.0	35.0	41.1	49.5		
Smoking History					<0.001	
Yes	38.5	39.6	41.6	54.9		
No	61.5	60.4	58.4	45.1		
MET^a^ Equivalent Physical Activity at Work (hrs/day)					< 0.001	
0	55.6	58.0	50.1	51.4		
0.01 to <8	18.0	13.5	14.2	11.5		
8 to <22	15.1	14.5	18.2	15.8		
>=22	11.2	14.0	17.5	21.2		
MET Equivalent Physical Activity Recreationally (hrs/day)					< 0.001	
0	35.3	44.3	47.2	55.7		
0.01 to <4	17.4	16.4	13.3	11.6		
4 to <8	17.6	14.4	13.6	12.0		
>=8	29.7	24.9	26.0			
Diabetes					<0.001	
Yes	12.7	11.2	9.9	7.4		
No	87.3	88.8	90.1	92.6		
Hypertension					0.119	
Yes	34.0	34.4	31.8	30.2		
No	66.0	65.6	68.2	69.8		
Total Energy Intake (kcal)	1925.5	2092.0	2156.9	2181.4	<0.001	

Unadjusted and adjusted odds ratios with 95% confidence intervals (CI) of moderate-to-severe depressive symptoms in varying CF ratio quartiles using univariate and multivariate models are shown in Table 2. Before adjustment, the odds of depressive symptoms significantly increased in Q4 of the CF ratio when compared to Q1 (OR 2.2, 95% CI 1.6-2.99). After adjustment, the odds of depressive symptoms remained significantly increased in Q4 of the CF ratio, when compared to Q1, albeit with a lower adjusted OR (adjOR 1.4, 95% CI 1.02-1.92). Incidentally, the odds of depressive symptoms were statistically significantly higher in the female gender, lower federal poverty ratios, non-married, smoking history, and hypertension. Females were 70% more likely to report depressive symptoms than men (adjOR 1.7, 95% CI 1.2-2.5). Participants with a federal poverty ratio of 1.30 and below had a 2.4-fold increase for reporting depressive symptoms than other socioeconomic groups (95% CI 1.7-3.3). Smokers were 90% more likely to show symptoms of depression than non-smokers (adjOR 1.9, 95% CI 1.3-2.6). Hypertensive participants had a 1.6-fold increase in depressive symptoms (95% CI 1.1-2.2) when compared with healthy adults. In contrast, when compared with all races, Mexicans (adjOR 0.6, 95% CI 0.4-0.9), non-Hispanic African Americans (adjOR 0.7, 95% CI 0.5-0.9), and non-Hispanic Asians (adjOR 0.5, 95% CI 0.3-0.9) all had a significantly decreased odds of depressive symptoms. Participants 60 years and older were 60% less likely to report depressive symptoms (adjOR 0.4, 95% CI 0.3-0.7). Physically active respondents who exercised at least four hours or more recreationally (MET equivalent) had a statistically significantly lower odds of depressive symptoms than sedentary respondents (adjOR 0.6, 95% CI 0.4-0.8). There was no statistically significant association between education level, BMI, alcohol intake, physical activity at work, and the presence of diabetes and depressive symptoms.

**Table 2:** Univariate and multivariate models assessing depression risk in varying carbohydrate-to-fiber ratios in the U.S. during 2013-2016. ^a^Odds ratio; ^b^Confidence interval; ^c^Reference group; ^d^This model adjusted for age, gender, race, education, BMI, alcohol consumption, poverty ratio, diabetes, hypertension, marital status, work and recreational physical activity, smoking, and total calorie intake; ^e^Rounded to hundredth place to show adjusted OR does not include 1.0; *p-value<0.05

	Unadjusted OR^a^ (95% CI^b^)	Adjusted OR^d^ (95% CI)
Carbohydrate-to-Fiber Ratio Quartiles		
Q1 2.3-12.1	Ref^c^	Ref
Q2 12.2-16.5	1.1 (0.8-1.5)	0.9 (0.6-1.3)
Q3 16.6-22.9	1.3 (1.0-1.9)	0.9 (0.6-1.4)
Q4 23.0-724.4	2.2 (1.6-2.99)*	1.4 (1.02-1.92)^e,^*
Age (years)		
18-39	Ref	Ref
40-59	1.2 (1.0-1.6)	0.9 (0.6-1.2)
60+	0.9 (0.7-1.3)	0.4 (0.3-0.7)*
Sex		
Male	Ref	Ref
Female	1.8 (1.4-2.3)*	1.7 (1.2-2.5)*
Race		
Non-Hispanic Whites	Ref	Ref
Mexican	0.9 (0.6-1.3)	0.6 (0.4-0.9)*
Other Hispanics	1.4 (1.0-1.9)	1.3 (0.8-2.0)
Non-Hispanic African Am.	1.0 (0.8-1.4)	0.7 (0.5-0.9)*
Non-Hispanic Asians	0.3 (0.2-0.6)*	0.5 (0.3-0.9)*
Other races	2.3 (1.3-3.9)*	1.9 (1.0-3.8)
Education Level		
Less than high school	1.4 (1.1-1.9)*	1.4 (0.9-2.1)
High school	Ref	Ref
More than high school	0.7 (0.5-0.9)*	1.0 (0.8-1.5)
Poverty Ratio		
1.30 and below	3.6 (2.7-4.7)*	2.4 (1.7-3.3)*
1.3-2.5	2.7 (2.0-3.7)*	2.0 (1.3-2.9)*
2.5-5.0	Ref	Ref
Marital Status		
Married	Ref	Ref
Non-married	2.1 (1.7-2.6)*	1.6 (1.3-2.1)*
N/A	1.0 (0.5-2.0)	1.2 (0.5-2.7)
BMI (kg/m^2^)		
Under 25	Ref	Ref
25 to <30	0.8 (0.6-1.1)	0.9 (0.7-1.2)
30+	1.5 (1.1-2.1)*	1.1 (0.8-1.4)
Alcohol Intake		
Less than 12 drinks/year	Ref	Ref
Non-heavy drinkers	0.9 (0.7-1.1)	1.3 (1.0-1.8)
Heavy drinkers	1.3 (1.0-1.6)*	1.3 (0.9-1.8)
Smoking History		
No	Ref	Ref
Yes	2.3 (1.8-3.0)*	1.9 (1.3-2.6)*
Physical Activity at Work (hrs/day)		
0	Ref	Ref
0.01 to <8	0.8 (0.5-1.1)	0.8 (0.5-1.1)
8 to <22	0.8 (0.6-1.0)	0.8 (0.5-1.1)
>=22	0.7 (0.5-0.9)*	0.7 (0.5-1.0)
Physical Activity Recreationally (hrs/day)		
0	Ref	Ref
0.01 to <4	0.8 (0.5-1.0)	1.1 (0.7-1.5)
4 to <8	0.4 (0.3-0.6)*	0.6 (0.4-0.8)*
>=8	0.3 (0.2-0.4)*	0.4 (0.3-0.5)*
Diabetes		
No	Ref	Ref
Yes	1.7 (1.3-2.2)*	1.4 (0.9-2.2)
Hypertension		
No	Ref	Ref
Yes	1.7 (1.4-2.2)*	1.6 (1.1-2.2)*
Total Energy Intake (kcal)	1.0 (0.9-0.9)*	1.0 (0.9-1.1)

## Discussion

This study revealed that the carbohydrate-to-fiber dietary intake ratio was positively associated with moderate-to-severe depressive symptoms. A higher carbohydrate-to-fiber dietary intake ratio increased the odds of depressive symptoms. In addition, individuals were less likely to report depressive symptoms if they were over the age of 60 years, identified as Mexican, Non-Hispanic African American, or Non-Hispanic Asian race, or got more than four hours per day of recreational physical activity.

These results are generally in line with the current scientific literature showing that higher levels of nutrient intake correlate with better mental health outcomes, and that fiber is inversely associated with the prevalence of depressive symptoms [10,11,16]. One randomized control trial (RCT) found that the intake of fiber was significantly lower in the depression group [20]. Additionally, previous studies have found that individuals consuming higher glycemic index (G.I.) carbohydrates have greater odds of depression or depressive symptoms [11,21]. While Meegan *et al.* found that individuals with high dietary quality were more likely to report well-being, no associations between any dietary measures and depressive symptoms were observed [9]. Finally, previous studies have shown that dietary fiber intake from vegetables and fruits was significantly inversely associated with depressive symptoms [10,11,16], but dietary intake of total and bread/cereal fiber has shown mixed results in relation to depressive symptoms [10,11,16]. These findings mirror the results of this study since bread and cereal sources of fiber tend to have higher CF ratios than vegetable sources of fibers, thereby reinforcing the importance of a balance between carbohydrates and fiber [22].

Recent evidence suggests that this relationship between carbohydrates and depression, and fiber and depression may be linked on a biochemical level via the inflammatory response. Patients with chronic inflammatory and autoimmune conditions are at a significantly higher risk of clinical depression [23-25]. On a cellular and molecular level, clinical depression is associated with increased cell-mediated immunity as well as increased oxidative and nitrosative stress, which induces chronic, low-grade inflammation and contributes to the progression of clinical depression [26,27]. Inflammatory mediators can alter brain signaling patterns affecting cognition and contribute to sickness behavior closely related to clinical depression and depression-like symptoms, including depressed mood, anhedonia, hyperalgesia, loss of appetite, fatigue, and cognitive deficits [23]. Together, these findings correlate systemic inflammation with the pathogenesis of depression. Therefore, it is imperative to identify modifiable mediators in these pathways that could ameliorate the symptoms of clinical depression and other related disease processes.

One area of emerging interest is intestinal dysbiosis induced by high-glycemic/high-carbohydrate food deficit in dietary fiber [7]. Fibers can regulate an anti-inflammatory response via Short Chain Fatty Acids (SCFAs), such as acetate, propionate, and butyrate, which are generated as byproducts of bacterial fermentation of prebiotics, i.e. fiber. Importantly the quantity and proportion of SCFAs depend on the quantity of prebiotic present [28]. Decreases in SCFAs, as found in a low fiber diet, lead to increased translocation of bacteria and pathogen-associated molecular patterns (PAMPs) like LPS across the intestinal epithelial barrier, which induces systemic inflammation [29-36]. SCFAs also directly regulate cell-mediated immune responses by acting as epigenetic regulators at tissues found throughout the body, including immune cells and the endothelium of the blood-brain barrier [37]. Conversely, they can also enhance an anti-inflammatory response by increasing induction of regulatory T-cell and interleukin (IL)-10 release while decreasing tumor necrosis factor (TNF)-α and interferon (IFN-γ release [38-41].

The secondary findings of this study further support these hypotheses. Notably, increased depressive symptoms were positively associated with an increased risk of developing clinical depression and mood symptoms [26,42]. Smoking and alcohol misuse have also been found to be significantly higher in those suffering from clinical depression [26]. This is noteworthy, as these substances are known to significantly increase systemic inflammation as well [43,44]. Meanwhile, decreased depressive symptoms were negatively associated with known reducers of inflammation and oxidative stress, such as recreational physical activity. Overall, these factors are related to the same pathway linking systemic inflammation and modulate clinical depression and depressive symptoms.

Naturally, this study has some limitations. Given the cross-sectional study design, causality cannot be determined. The PHQ-9 uses criteria from DSM-V in order to detect and determine severity of depressive symptoms, making it a widely used tool [15]. Since the variable was analyzed dichotomously, participants who suffer from severe depressive symptoms and participants who have moderate symptoms were analyzed in the same group, making the variable prone to misclassification bias. The self-reporting nature of this variable could have also impacted the results; due to the cultural stigma of depression, there could be underreporting by the participants. In the same vein, this limits external validity to a population that is culturally similar to the United States. Additionally, dietary intake information was based on a 24-hour dietary recall interview, which is prone to interviewer and recall bias. The usage of antidepressants was not available in the database and could pose as a confounding variable. Last, any inherent limitations or errors in the collection and distribution of the NHANES data would be inherited in this study.

## Conclusions

In conclusion, given the connections between dietary fiber and carbohydrates in inducing systemic inflammation and the bi-directional link between inflammation and depressive symptoms, a metric combining these factors may be helpful in identifying a key aspect of dietary risk for depressive symptoms. The results of this study support this hypothesis, finding that participants with increasing carbohydrate-to-fiber ratios may be more likely to report moderate-to-severe depressive symptoms. Additionally, it is well established in previous literature that participants reporting modifiable factors such as sedentary lifestyles, smoking, and heavy alcohol intake are more likely to experience depressive symptoms. Reduction in these activities, in conjunction with dietary carbohydrates to fiber ratio that falls within quartiles 1 and 2, may lead to a decrease in depressive symptoms. These are promising findings as dietary intake is a readily modifiable lifestyle factor. Using a patient’s carbohydrate-to-fiber ratio may give both clinicians and patients better insight into the associations between diet and clinical depression. For clinicians offering dietary counseling, it would be beneficial to all patients, especially those with moderate depressive symptoms, treatment-resistant depression, and those opposed to medications. Future studies should further examine the links between dietary-induced inflammation and other conditions, including bipolar disorder, anxiety, and schizophrenia. Concerning clinical depression, future studies could analyze PHQ-9 scores as a categorical variable and determine the effect of increasing carbohydrate-to-fiber ratios on the severity of depressive symptoms. Since the CF ratio is a new marker, further prospective studies are needed to validate it as a dietary measurement.

## References

[1] Brody Dj PLAHJ (2021). Prevalence of Depression Among Adults Aged 20 and Over: United States, 2013-2016. https://www.cdc.gov/nchs/data/databriefs/db303.pdf.

[2] Lépine JP, Briley M (2011). The increasing burden of depression. Neuropsychiatr Dis Treat.

[3] Eshraghi RS, Deth RC, Mittal R, Aranke M, Kay SS, Moshiree B, Eshraghi AA (2018). Early disruption of the microbiome leading to decreased antioxidant capacity and epigenetic changes: implications for the rise in autism. Front Cell Neurosci.

[4] Bach Knudsen KE, Lærke HN, Hedemann MS (2018). Impact of diet-modulated butyrate production on intestinal barrier function and inflammation. Nutrients.

[5] Matt SM, Allen JM, Lawson MA, Mailing LJ, Woods JA, Johnson RW (2018). Butyrate and dietary soluble fiber improve neuroinflammation associated with aging in mice. Front Immunol.

[6] Delzenne NM, Neyrinck AM, Cani PD (2013). Gut microbiota and metabolic disorders: how prebiotic can work?. Br J Nutr.

[7] Spreadbury I (2012). Comparison with ancestral diets suggests dense acellular carbohydrates promote an inflammatory microbiota, and may be the primary dietary cause of leptin resistance and obesity. Diabetes Metab Syndr Obes.

[8] Davison KM, Kaplan BJ (2012). Nutrient intakes are correlated with overall psychiatric functioning in adults with mood disorders. Can J Psychiatry.

[9] Meegan AP, Perry IJ, Phillips CM (2017). The association between dietary quality and dietary guideline adherence with mental health outcomes in adults: a cross-sectional analysis. Nutrients.

[10] Miki T, Eguchi M, Kurotani K (2016). Dietary fiber intake and depressive symptoms in Japanese employees: The Furukawa Nutrition and Health Study. Nutrition.

[11] Gopinath B, Flood VM, Burlutksy G, Louie JC, Mitchell P (2016). Association between carbohydrate nutrition and prevalence of depressive symptoms in older adults. Br J Nutr.

[12] (2020). Mental Health - Depression Screener. U.S. Department of Health and Human Services, Centers for Disease Control and Prevention. https://wwwn.cdc.gov/Nchs/Nhanes/2015-2016/DPQ_I.htm.

[13] (2020). National Health and Nutrition Examination Survey. https://www.cdc.gov/nchs/nhanes/index.htm.

[14] (2021). Department of Health and Human Services and U.S. Department of Agriculture 2015-2020 Dietary Guidelines for Americans. Agriculture.

[15] Kroenke K, Spitzer RL, Williams JB (2001). The PHQ-9: validity of a brief depression severity measure. J Gen Intern Med.

[16] Xu H, Li S, Song X, Li Z, Zhang D (2018). Exploration of the association between dietary fiber intake and depressive symptoms in adults. Nutrition.

[17] Falk G, Aussenberg RA (2021). The Supplemental Nutrition Assistance Program (SNAP): Categorical Eligibility. https://crsreports.congress.gov/product/pdf/R/R42054.

[18] NCHS) CfDCaPCNCfHS. Body Mass Index . https://www.cdc.gov/healthyweight/assessing/bmi/index.html (2021). Body Mass Index. https://www.cdc.gov/healthyweight/assessing/bmi/index.html..

[19] Menke A, Casagrande S, Geiss L, Cowie CC (2015). Prevalence of and trends in diabetes among adults in the United States, 1988-2012. JAMA.

[20] Kaner G, Soylu M, Yüksel N, Inanç N, Ongan D, Başmısırlı E (2015). Evaluation of nutritional status of patients with depression. Biomed Res Int.

[21] Haghighatdoost F, Azadbakht L, Keshteli AH (2016). Glycemic index, glycemic load, and common psychological disorders. Am J Clin Nutr.

[22] Opie RS, O'Neil A, Jacka FN, Pizzinga J, Itsiopoulos C (2018). A modified Mediterranean dietary intervention for adults with major depression: dietary protocol and feasibility data from the SMILES trial. Nutr Neurosci.

[23] Amodeo G, Trusso MA, Fagiolini A (2017). Depression and inflammation: disentangling a clear yet complex and multifaceted link. Neuropsychiatry.

[24] Lee CH, Giuliani F (2019). The role of inflammation in depression and fatigue. Front Immunol.

[25] Hashioka S, Inoue K, Hayashida M, Wake R, Oh-Nishi A, Miyaoka T (2018). Implications of systemic inflammation and periodontitis for major depression. Front Neurosci.

[26] Berk M, Williams LJ, Jacka FN (2013). So depression is an inflammatory disease, but where does the inflammation come from?. BMC Med.

[27] Beydoun MA, Obhi HK, Weiss J, Canas JA, Beydoun HA, Evans MK, Zonderman AB (2020). Systemic inflammation is associated with depressive symptoms differentially by sex and race: a longitudinal study of urban adults. Mol Psychiatry.

[28] Wong JM, de Souza R, Kendall CW, Emam A, Jenkins DJ (2006). Colonic health: fermentation and short chain fatty acids. J Clin Gastroenterol.

[29] Willemsen LE, Koetsier MA, van Deventer SJ, van Tol EA (2003). Short chain fatty acids stimulate epithelial mucin 2 expression through differential effects on prostaglandin E(1) and E(2) production by intestinal myofibroblasts. Gut.

[30] Barcelo A, Claustre J, Moro F, Chayvialle JA, Cuber JC, Plaisancié P (2000). Mucin secretion is modulated by luminal factors in the isolated vascularly perfused rat colon. Gut.

[31] Jung TH, Park JH, Jeon WM, Han KS (2015). Butyrate modulates bacterial adherence on LS174T human colorectal cells by stimulating mucin secretion and MAPK signaling pathway. Nutr Res Pract.

[32] Iacob S, Iacob DG (2019). Infectious threats, the intestinal barrier, and its Trojan horse: dysbiosis. Front Microbiol.

[33] Zheng L, Kelly CJ, Battista KD (2017). Microbial-derived butyrate promotes epithelial barrier function through IL-10 receptor-dependent repression of claudin-2. J Immunol.

[34] Yan H, Ajuwon KM (2017). Butyrate modifies intestinal barrier function in IPEC-J2 cells through a selective upregulation of tight junction proteins and activation of the Akt signaling pathway. PLoS One.

[35] Miao W, Wu X, Wang K (2016). Sodium butyrate promotes reassembly of tight junctions in caco-2 monolayers involving inhibition of MLCK/MLC2 pathway and phosphorylation of PKCβ2. Int J Mol Sci.

[36] Tong LC, Wang Y, Wang ZB (2016). Propionate ameliorates dextran sodium sulfate-induced colitis by improving intestinal barrier function and reducing inflammation and oxidative stress. Front Pharmacol.

[37] Cryan JF, O'Riordan KJ, Cowan CS (2019). The microbiota-gut-brain axis. Physiol Rev.

[38] Cox MA, Jackson J, Stanton M (2009). Short-chain fatty acids act as antiinflammatory mediators by regulating prostaglandin E(2) and cytokines. World J Gastroenterol.

[39] Smith PM, Howitt MR, Panikov N (2013). The microbial metabolites, short-chain fatty acids, regulate colonic Treg cell homeostasis. Science.

[40] Nastasi C, Candela M, Bonefeld CM (2015). The effect of short-chain fatty acids on human monocyte-derived dendritic cells. Sci Rep.

[41] Lührs H, Gerke T, Müller JG (2002). Butyrate inhibits NF-kappaB activation in lamina propria macrophages of patients with ulcerative colitis. Scand J Gastroenterol.

[42] Möller M, Du Preez JL, Viljoen FP, Berk M, Emsley R, Harvey BH (2013). Social isolation rearing induces mitochondrial, immunological, neurochemical and behavioural deficits in rats, and is reversed by clozapine or N-acetyl cysteine. Brain Behav Immun.

[43] Nunes SO, Vargas HO, Brum J (2012). A comparison of inflammatory markers in depressed and nondepressed smokers. Nicotine Tob Res.

[44] Wang HJ, Zakhari S, Jung MK (2010). Alcohol, inflammation, and gut-liver-brain interactions in tissue damage and disease development. World J Gastroenterol.

